# Larotrectinib induces autophagic cell death through AMPK/mTOR signalling in colon cancer

**DOI:** 10.1111/jcmm.17530

**Published:** 2022-10-17

**Authors:** Wencheng Kong, Hangzhang Zhu, Sixing Zheng, Guang Yin, Panpan Yu, Yuqiang Shan, Xinchun Liu, Rongchao Ying, Hong Zhu, Shenglin Ma

**Affiliations:** ^1^ Zhejiang Province Key Laboratory of Anti‐cancer Drug Research, Institute of Pharmacology and Toxicology, College of Pharmaceutical Sciences Zhejiang University Hangzhou China; ^2^ Department of Gastroenterological Surgery, Affiliated Hangzhou First People's Hospital Zhejiang University School of Medicine Hangzhou China; ^3^ Translational Medicine Research Center, Key Laboratory of Clinical Cancer Pharmacology and Toxicology Research of Zhejiang Province, Affiliated Hangzhou First People's Hospital Zhejiang University School of Medicine Hangzhou China

**Keywords:** AMPK/mTOR signalling, autophagy flux, colon cancer, epithelial–mesenchymal transition, Larotrectinib

## Abstract

Larotrectinib (Lar) is a highly selective and potent small‐molecule inhibitor used in patients with tropomyosin receptor kinase (TRK) fusion‐positive cancers, including colon cancer. However, the underlying molecular mechanisms specifically in patients with colon cancer have not yet been explored. Our data showed that Lar significantly suppressed proliferation and migration of colon cancer cells. In addition, Lar suppressed the epithelial–mesenchymal transition (EMT) process, as evidenced by elevation in E‐cadherin (E‐cad), and downregulation of vimentin and matrix metalloproteinase (MMP) 2/9 expression. Furthermore, Lar was found to activate autophagic flux, in which Lar increased the ratio between LC3II/LC3I and decreased the expression of p62 in colon cancer cells. More importantly, Lar also increased AMPK phosphorylation and suppressed mTOR phosphorylation in colon cancer cells. However, when we silenced AMPK in colon cancer cells, Lar‐induced accumulation of autolysomes as well as Lar‐induced suppression of the EMT process were significantly diminished. An in vivo assay also confirmed that tumour volume and weight decreased in Lar‐treated mice than in control mice. Taken together, this study suggests that Lar significantly suppresses colon cancer proliferation and migration by activating AMPK/mTOR‐mediated autophagic cell death.

## 1 INTRODUCTION

Colon cancer (CC) is a common gastrointestinal malignant tumour and the third leading cause of cancer‐related morbidity and mortality.^1^ Epithelial–mesenchymal transition (EMT), a process in which immobile epithelial cells are transformed into mobile mesenchymal cells, is tightly associated with cancer progression and metastasis.^2^, ^3^ During the EMT progress, epithelial marker proteins such as E‐cadherin (E‐cad) are reduced in cancer cells.^1^ In contrast, an elevation in mesenchymal features of proteins such as vimentin and matrix metalloproteinase (MMP)2/9 has been observed in cancer cells.^4^ Hence, the mechanisms by which EMT is suppressed may have a great potential in the treatment of CC.

Autophagy is a conserved process that plays a key role in maintaining the balance between cell death and survival.^5^, ^6^ Typical features of autophagic cell death include the accumulation of autophagic organelles such as autophagosomes and autolysomes.^7^ Light chain 3‐II (LC3‐II) is an autophagy marker that plays a key role in the elongation of double membranes during induction of autophagic flux and is necessary for the formation of autophagosomes.^7^ p62/SQSTM1 is another key autophagy marker that reduces polyubiquitinated substrates via autophagy flux, resulting in its own degradation.^8^ In many circumstances, basal autophagy maintains cell survival by degrading dysfunctional organelles and long‐life cytoplasmic proteins, whereas excessive autophagy results in autophagic cell death and subsequent cancer cell elimination.^5^, ^8^

Larotrectinib (Lar) is a highly selective and potent small‐molecule inhibitor of all three tropomyosin receptor kinase (TRK) proteins, TRKA, TRKB and TRKC, in patients with TRK fusion‐positive cancer, including colon cancer.^9^ TRK is shown to be involved in the process of autophagy related to the nervous system.^10^, ^11^ For instance, brain‐derived neurotrophic factor (BDNF) signalling inhibits autophagy via the TRKB pathway in vivo.^10^ However, whether Lar suppresses the development of CC via autophagy has not yet been explored.

## 2 RESULTS

### 2.1 Lar suppressed colon cancer cell survival and migration

**FIGURE 1:**
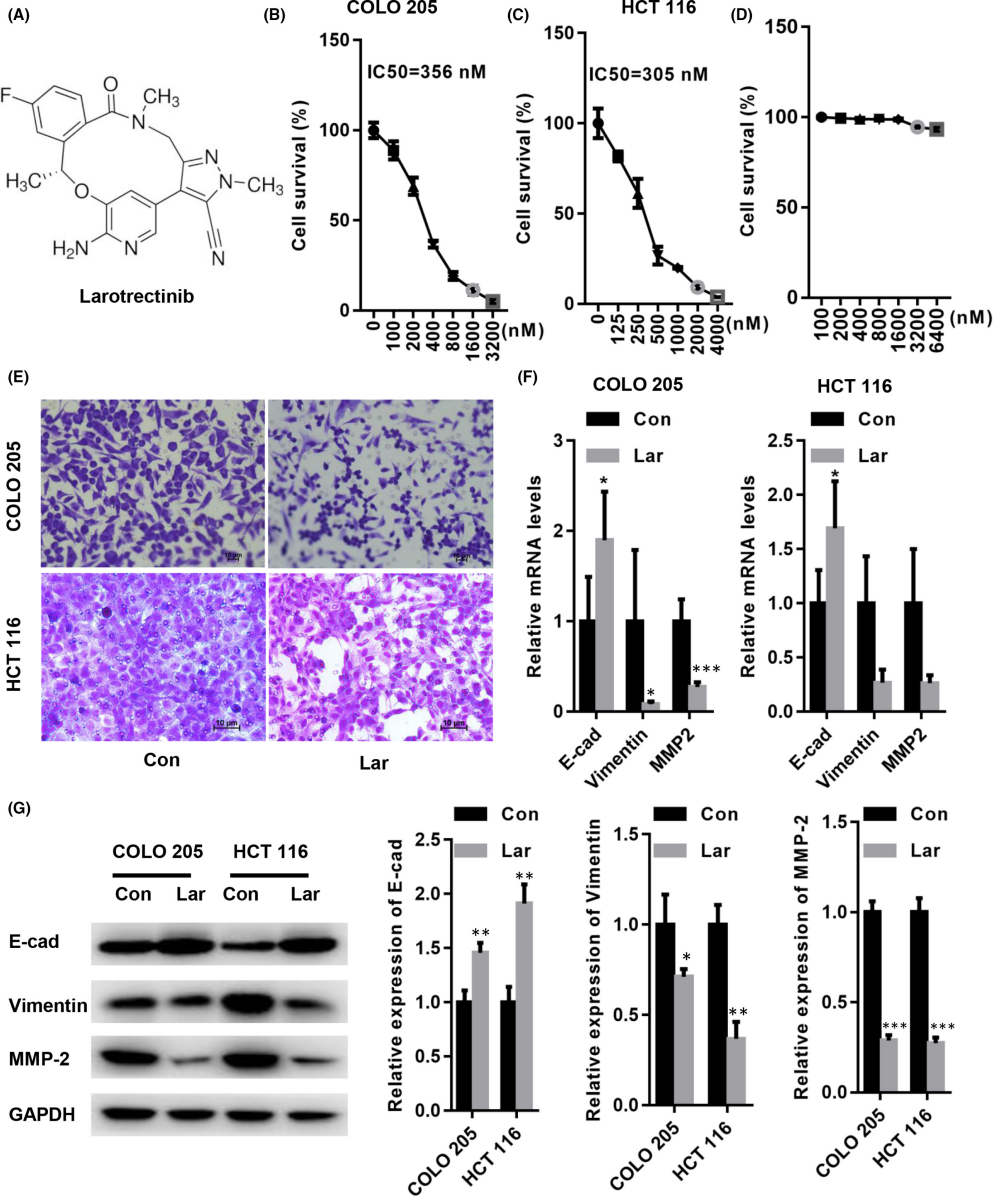
Lar suppressed colon cancer cell survival and migration. (A) Larotrectinib (Lar). CCK‐8 assay was carried out to determine the IC50 of Lar in COLO205 (B), HCT116 (C) and human colonic mucosal epithelial cells (D). (E) Transwell assay demonstrated that Lar decreased the migratory capacity of COLO205 and HCT116 cells. (F) RT‐PCR analysis showed that Lar elevated E‐cad mRNA level but decreased vimentin and MMP2 mRNA levels in COLO205 and HCT116 cells. (G) Western blot assay showed that Lar increased the expression of E‐cad but reduced the expression of vimentin and MMP2 in COLO205 and HCT116 cells. *p < 0.05, **p < 0.01, ***p < 0.001 vs. control; *n* = 3 independent repeats apart from replicates.

We first investigated the effects of Lar on survival rate of colon cancer cells. CCK‐8 assay showed that the survival rates of COLO205 and HCT116 cells were significantly suppressed by Lar treatment (Figure 1A–C). The IC50 values for COLO205 and HCT116 cells were 356 and 305 nM, respectively. In the subsequent experiments, 300 nM Lar was used. However, Lar did not significantly suppress the viability of Human colonic mucosal epithelial cells (Figure 1D). A transwell assay was used to determine the migratory capacity induced by Lar, and our data revealed that Lar treatment significantly suppressed the migratory capacity of COLO205 and HCT116 cells (Figure 1E). Because EMT is known to play an important role in colon cancer cell migration, we explored the changes in EMT markers after Lar treatment. RT‐PCR analysis showed that the mRNA level of E‐cad was significantly increased, while the mRNA levels of vimentin and MMP2 were decreased in COLO205 and HCT116 cells treated with Lar compared with that of control (Figure 1F). As shown in Figure 1E, Lar significantly upregulated the expression of the epithelial marker E‐cad but reduced the expression of mesenchymal markers, including vimentin, and MMP2 (Figure 1G). These data indicate that Lar acts as a tumour suppressor in colon cancer cells.

### 2.2 Lar enhanced autophagic flux in COLO205 and HCT116 cells

**FIGURE 2:**
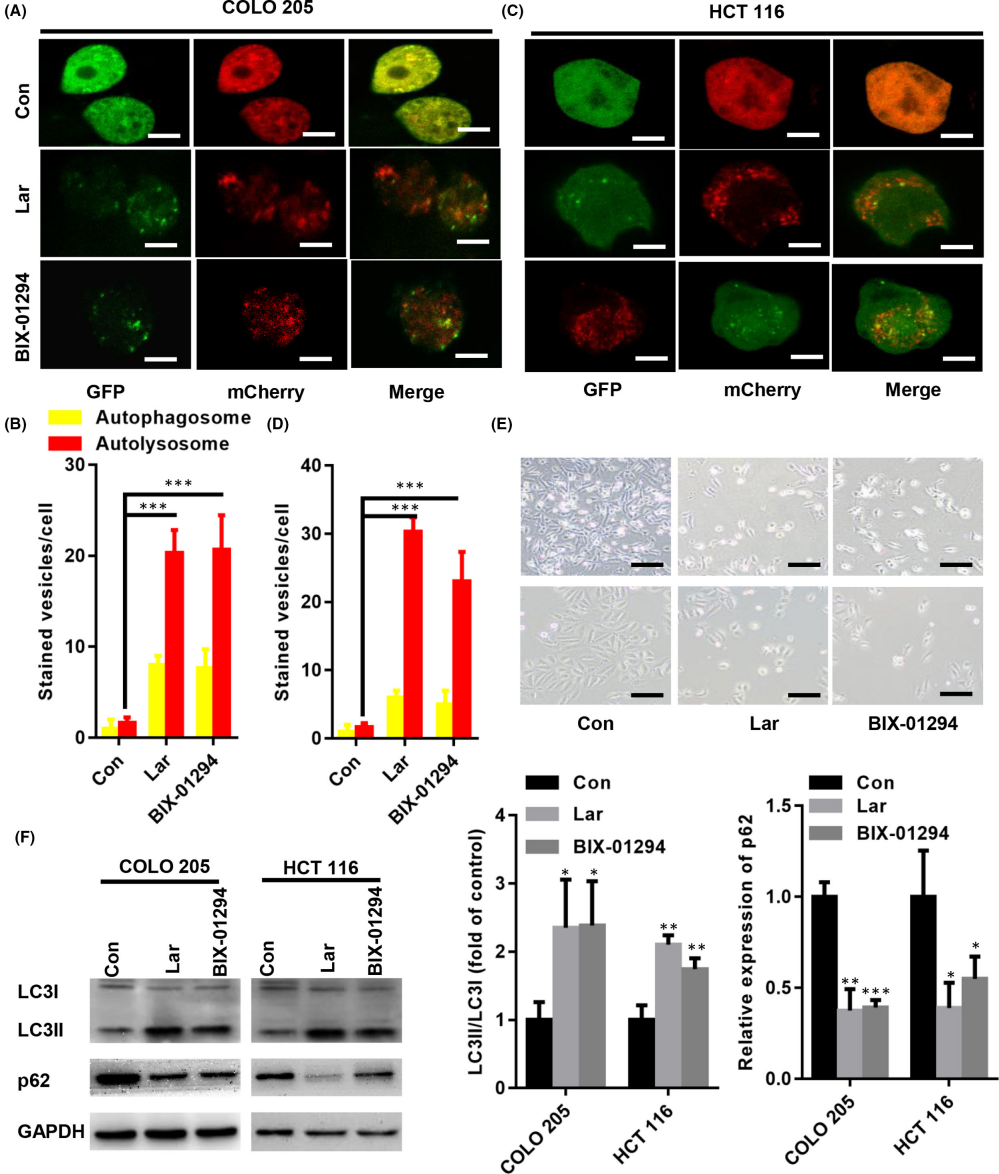
Lar enhanced autophagic flux in COLO205 and HCT116 cells. Lar or BIX‐01294 induced a higher number of red fluorescent puncta than yellow puncta in COLO205 (A, B) and HCT116 (C, D) cells (Bar = 10 μm). (E) Under bright field, compared with control, both of Lar or BIX‐01294 reduced COLO205 and HCT116 cell number and led to shrink of COLO205 and HCT116 cell morphology (Bar = 50 μm). (F) Western blot analysis demonstrated that LC3II/LC3I was significantly elevated in Lar‐treated or BIX‐01294‐treated COLO205 and HCT116 cells, whereas the expression of p62 was decreased. *p < 0.05, **p < 0.01, ***p < 0.001 vs. control; *n* = 3 independent repeats apart from replicates.

To compare the Lar mediated anti‐tumour effect is protective or cell death mechanism, BIX‐01294, a strong autophagy inducer, which promotes autophagy‐associated cell death in colon cancer cells,^12^ was selected in the present study. As shown in Figure 2A–D, both the yellow and red fluorescent puncta were increased in Lar‐treated or BIX‐01294‐treated COLO205 and HCT116 cells. More importantly, Lar or BIX‐01294 induced a higher number of red fluorescent puncta than yellow puncta in COLO205 and HCT116 cells (Figure 2A–D). We also compared the cell proliferation and morphology changes of Lar and BIX‐01294 in COLO205 and HCT116 cells. Our data showed that compared with control, both of Lar or BIX‐01294 reduced COLO205 and HCT116 cell number and led to shrink of COLO205 and HCT116 cell morphology (Figure 2E). Western blot analysis demonstrated that LC3II/LC3I was significantly elevated in Lar‐treated or BIX‐01294‐treated COLO205 and HCT116 cells, whereas the expression of p62 was decreased (Figure 2F). Taken together, these data indicate that Lar enhances autophagy‐associated cell death in colon cancer cells.

### 2.3 Chloroquine (CQ) blocked Lar‐induced autophagic flux in colon cancer cells

**FIGURE 3:**
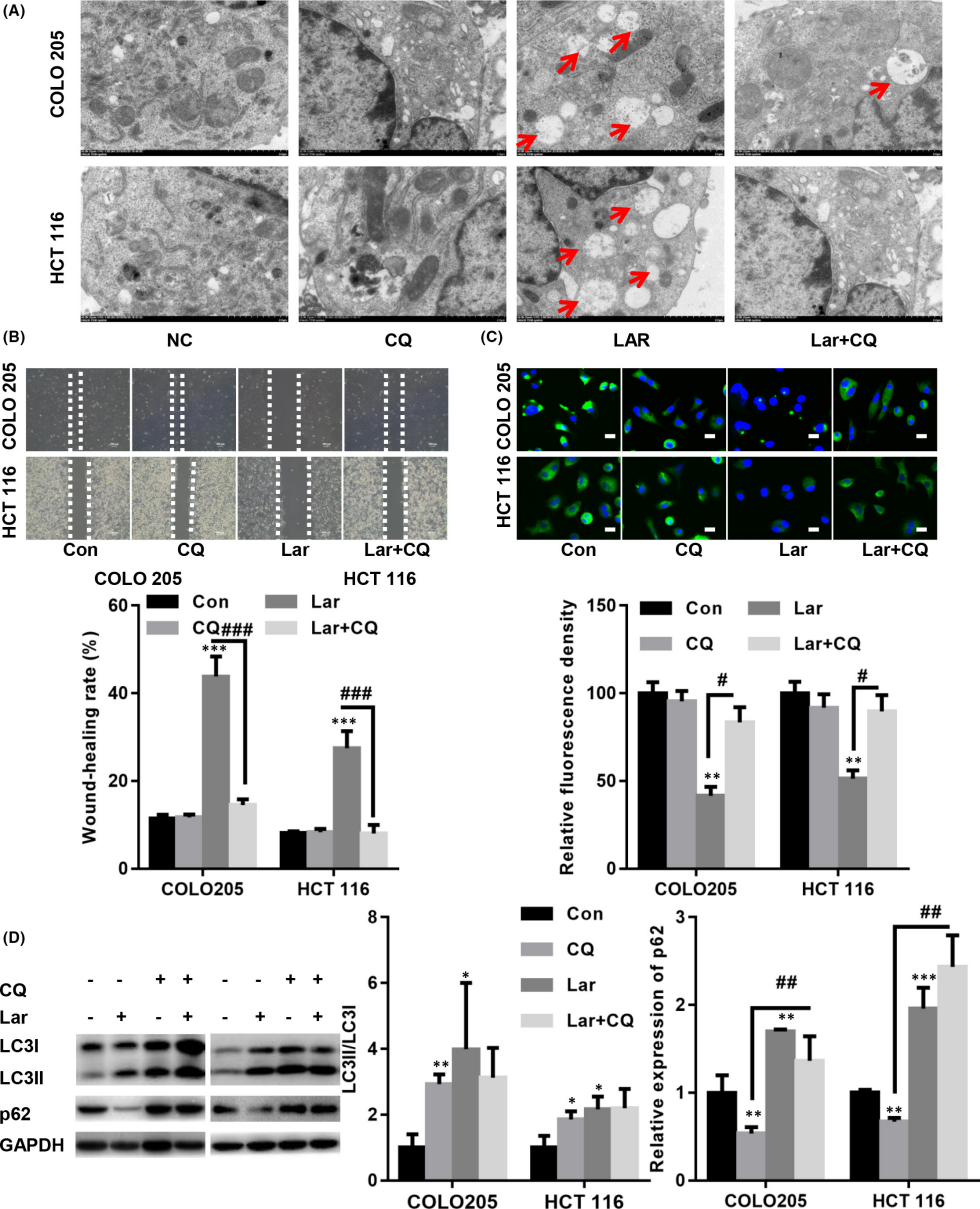
Chloroquine (CQ) blocked Lar‐induced autophagic flux in COLO205 and HCT116 cells. COLO205 and HCT116 cells were pre‐treated with 20 μM CQ for 2 h at 37°C and were further treated with or without 300 nM Lar for 24 h at 37°C. (A) Representative images of TEM (autolysomes are indicated by red arrows). (B) Wound healing assay revealed that Lar decreased the migratory capacity in COLO205 and HCT116 cells; however, pre‐incubation with CQ blocked such effects. (C) IF staining showed that Lar decreased the fluorescence intensity of Ki‐67, but pre‐incubation with CQ partially reversed such effects (magnification 20×). (D) Western blot analysis showed that Lar‐induced downregulation of p62 was abolished by CQ, while no significant changes in LC3II/LC3I ratio was observed in COLO205 and HCT116 cells. **p < 0.01, ***p < 0.001 vs. as indicated; *n* = 3 independent repeats apart from replicates.

To test whether Lar induces tumour suppression in colon cancer cells by enhancing autophagic flux, COLO205 and HCT116 cells were treated with 20 μM CQ, which suppressed autophagosome‐lysosome fusion. When the cells were co‐treated with CQ and Lar, the number of autolysosomes decreased in colon cancer cells compared with those of CQ or Lar alone (Figure 3A). Meanwhile, wound healing assay indicated that Lar decreased the migratory capacity of COLO205 and HCT116 cells: however, pre‐incubation with CQ blocked these effects (Figure 3B). Immunofluorescence staining showed that treatment with Lar significantly reduced the relative fluorescence intensity of Ki‐67, an important proliferation marker; however, pre‐incubation with CQ partially reversed these effects (Figure 3C). Similarly, Western blot assay showed that after CQ treatment, the ratio between LC3II/LC3I was increased and the expression of p62 was also elevated in COLO205 and HCT116 cells (Figure 3D). Moreover, Lar‐induced downregulation of p62 was abolished by CQ, while no significant changes in LC3II/LC3I ratio was observed in COLO205 and HCT116 cells (Figure 3D). These observations indicated that Lar‐induced inhibitory effects on colon cancer cells were mediated by the induction of autophagic flux.

### 2.4 Lar elevated autophagy via AMPK/mTOR signalling in colon cancer cells

**FIGURE 4:**
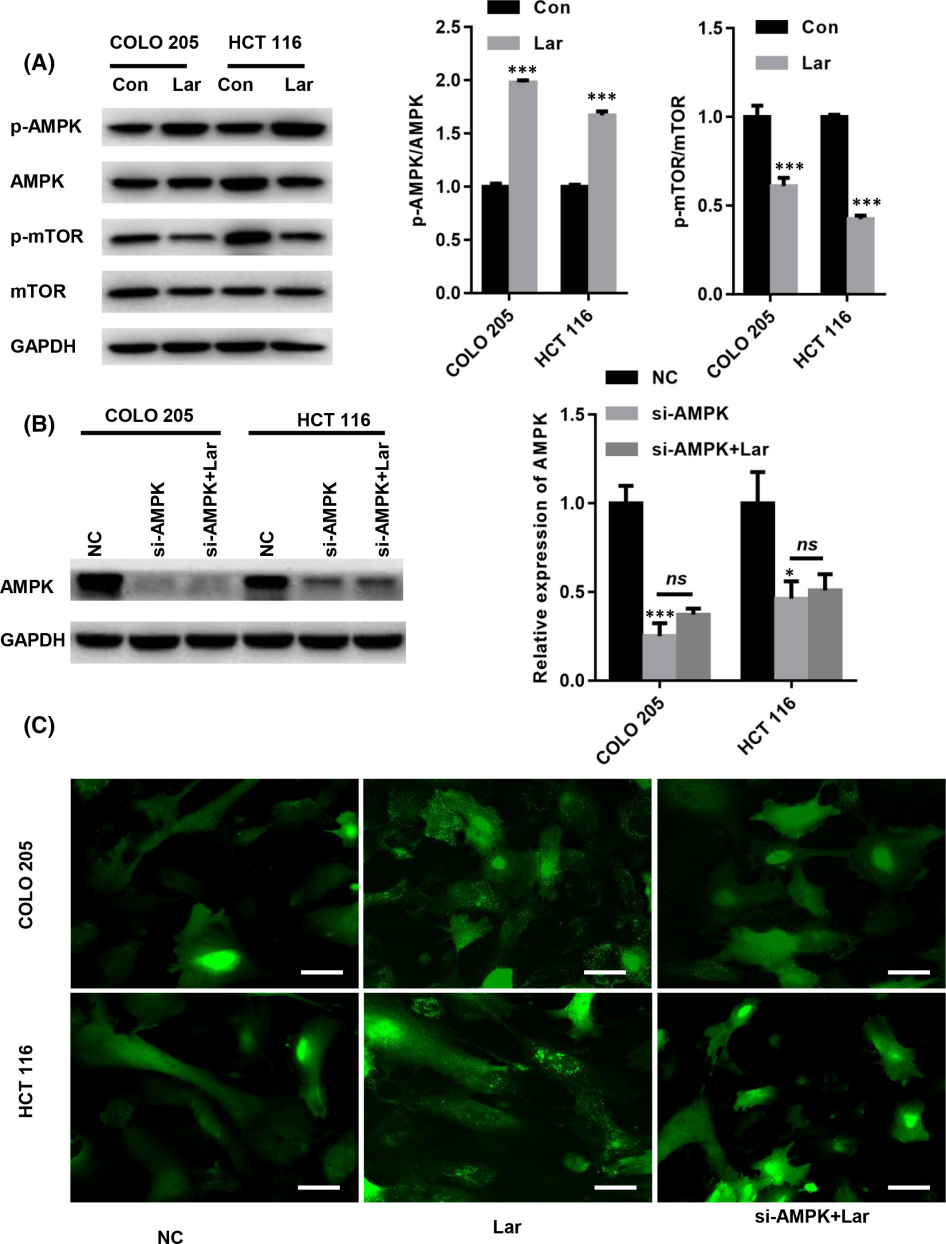
Lar elevated autophagy via AMPK/mTOR signalling in colon cancer cells. (A) Western blot analysis demonstrated that treatment with Lar significantly elevated AMPK phosphorylation and suppressed mTOR phosphorylation in COLO205 and HCT116 cells. (B) AMPK siRNA significantly repressed AMPK expression in COLO205 and HCT116 cells with or without Lar treatment. (C) Representative images of LC3 puncta in COLO205 and HCT116 cells treated with Lar and/or siAMPK (Bar = 10 μm). *p < 0.05, ***p < 0.001 vs. as indicated; *n* = 3 independent repeats apart from replicates.

Studies have shown that the AMP‐activated protein kinase (AMPK)/mechanistic target of rapamycin (mTOR) signalling pathway is an important regulator in triggering autophagic cell death.^13^, ^14^ Hence, we explored the effects of Lar on AMPK/mTOR signalling. Western blot analysis demonstrated that Lar treatment significantly elevated AMPK phosphorylation and suppressed mTOR phosphorylation in COLO205 and HCT116 cells (Figure 4A). To confirm whether Lar elevates autophagy via AMPK/mTOR signalling via AMPK, a specific siRNA that targets AMPK was selected. As shown in Figure 4B, AMPK siRNA significantly repressed AMPK expression in COLO205 and HCT116 cells with or without Lar treatment (Figure 4B). GFP‐LC3 transfection assay showed that Lar significantly increased LC3 puncta in COLO205 and HCT116 cells (Figure 4C). In contrast, the siRNA targeting AMPK abolished accumulation of LC3 puncta in Lar‐treated COLO205 and HCT116 cells (Figure 4C). These data indicate that ablation of AMPK decreases Lar‐induced lysosome accumulation in COLO205 and HCT116 cells.

### 2.5 Knockdown of AMPK abolished Lar‐induced suppression of EMT in colon cancer cells

**FIGURE 5:**
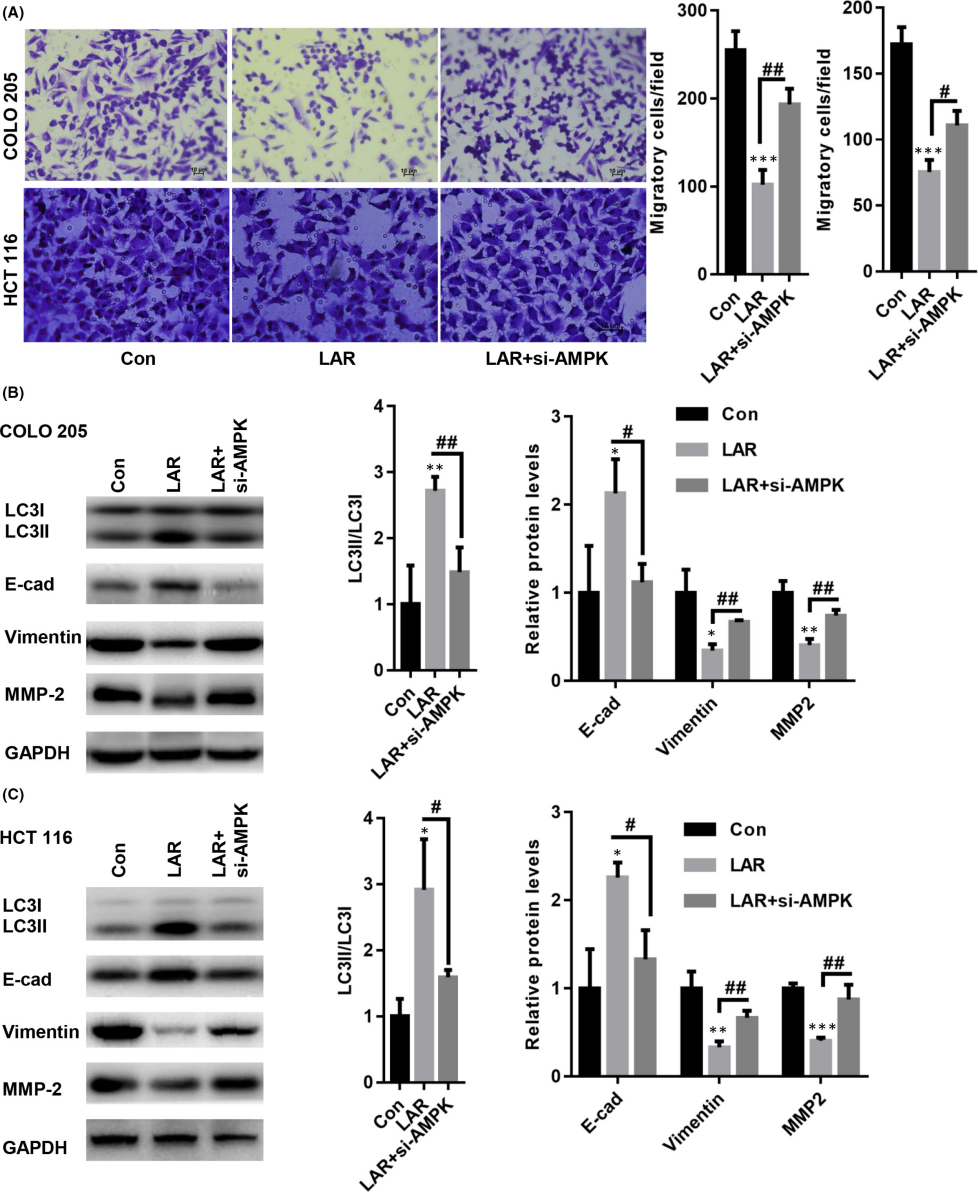
Knockdown of AMPK abolished Lar‐induced suppression of EMT in colon cancer cells. (A) Transwell assay showed that Lar decreased COLO205 and HCT116 cell migration; however, siAMPK marginally alleviated Lar‐induced migration inhibition in COLO205 and HCT116 cells. Lar‐induced upregulation of E‐cad and downregulation of vimentin and MMP2 were observed to be partially restored by siAMPK in COLO205 (B) and HCT116 (C) cells. *p < 0.05, ***p < 0.001 vs. Con; #*p* < 0.05, ##*p* < 0.01 vs. as indicated; *n* = 3 independent repeats apart from replicates.

Next, we determined whether silencing of AMPK (siAMPK) reversed Lar‐induced suppression of EMT in colon cancer cells. A transwell assay showed that Lar decreased COLO205 and HCT116 cell migration; however, siAMPK marginally alleviated Lar‐induced migration inhibition in COLO205 and HCT116 cells (Figure 5A). Besides, the elevated LC3II/LC3I ratio induced by Lar was also decreased by siAMPK transfection in COLO205 and HCT116 cells (Figure 5B,C). Furthermore, Lar‐induced upregulation of E‐cad and downregulation of vimentin and MMP2 were partially restored by siAMPK in COLO205 and HCT116 cells (Figure 5B,C). From these data, we can conclude that AMPK signalling contributes to Lar‐induced enhancement of autophagy.

### 2.6 Lar decreased in vivo tumour growth

**FIGURE 6:**
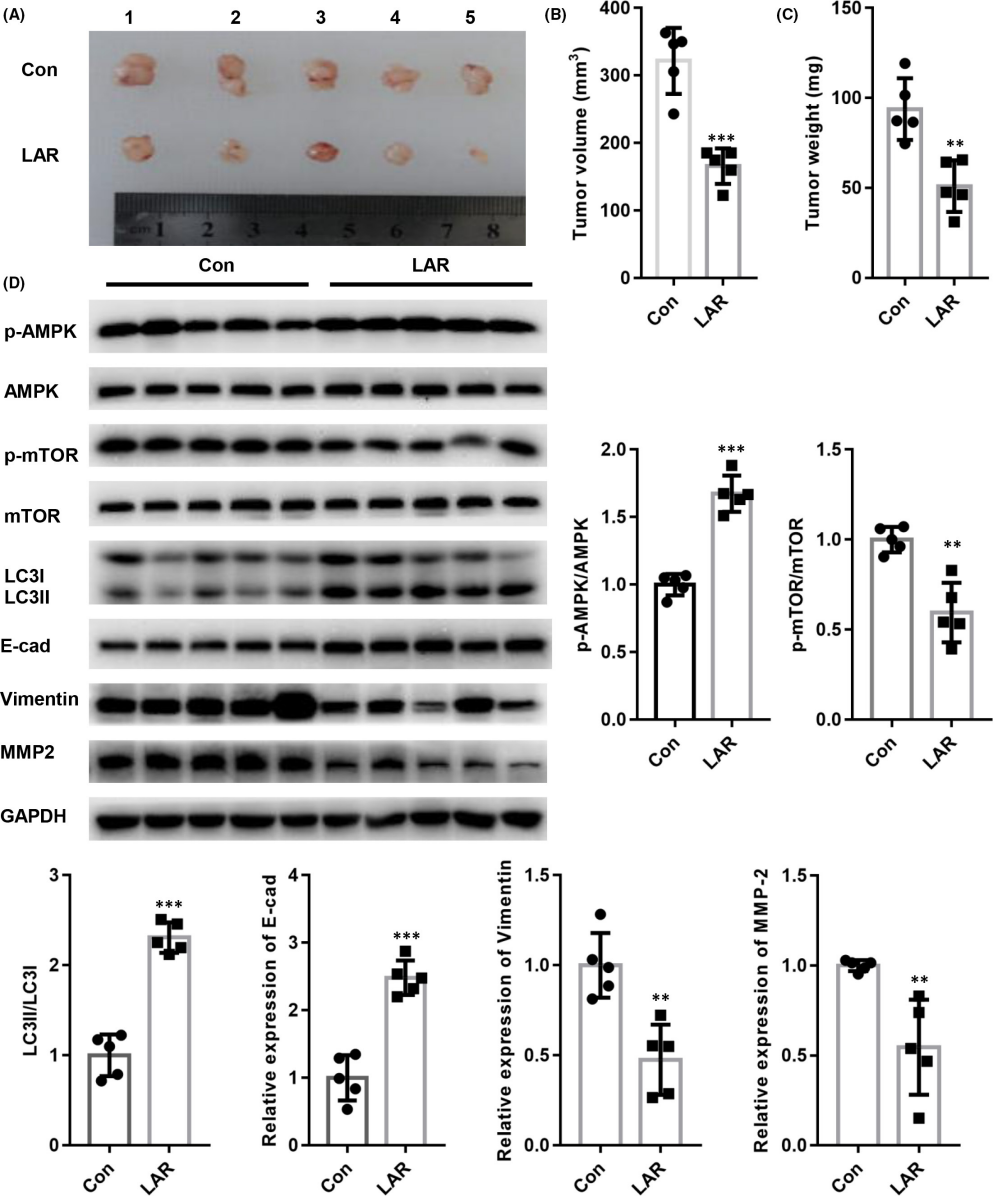
Lar decreased tumour growth in vivo. (A) Representative tumour images. Lar significantly reduced tumour volume (B) and weight (C) of HCT116 cells compared with those of control. (D) Western blot analysis of AMPK/mTOR signalling in tumour tissues after Lar treatment. **p < 0.01, ***p < 0.001 vs. Con; *n* = 5 for each group.

We investigated whether Lar exhibited anti‐cancer activity in vivo. Three weeks after Lar treatment, the tumour volume and weight of HCT116 cells were significantly reduced compared with those of control (Figure 6A–C). Consistent with the in vitro findings, Lar increased AMPK phosphorylation and decreased mTOR phosphorylation in vivo (Figure 6D). Lar treatment also increased the LC3II/LC3I ratio in vivo (Figure 6D). Furthermore, Lar elevated the expression of E‐cad, but reduced the expression of vimentin and MMP2 in vivo (Figure 6D). Taken together, these results indicate the anti‐cancer effects of LAR in colon cancer in vivo.

## 3 DISCUSSION

Autophagy is a highly conserved self‐degradation process by means of which unnecessary intracellular components are degraded in autophagolysosomes or recycled.^15^ Autophagy is a double‐edged sword.^16^ On the one hand, it protects cells from various cellular stresses; on the other hand, it triggers cell death independent of apoptosis, known as autophagic cell death.^16^ As an oral and highly selective pan‐TRK inhibitor, Lar is widely accepted to treat paediatric and adult patients with advanced or metastatic solid tumours, including colon cancer.^9^ However, the specific mechanism underlying the role of Lar in the treatment of colon cancer remains unclear.

In this study, we demonstrated that anti‐cancer effects of Lar were accompanied by the induction of autophagy in colon cancer cells. At a concentration of 300 nM, Lar significantly suppressed the proliferation and migration of COLO205 and HCT116 cells. EMT is a process that transforms immobile epithelial cells into mobile mesenchymal cells to subsequently promote cancer invasion and metastasis.^17^ Hence, we explored whether Lar induces changes in the EMT of colon cancer cells. We confirmed that Lar suppressed the EMT process, along with the elevation of E‐cad expression and downregulation of vimentin and MMP‐2 expression.

Autophagy is a major process that contributes to the maintenance of intracellular energy metabolism and self‐survival.^18^ Recently, it has been demonstrated that autophagy and EMT are tightly correlated in regulating tumorigenesis and tumour progression.^19^, ^20^ During the initial stage of tumour, autophagy suppresses cancer invasion and metastasis by selectively degrading important signalling molecules in the EMT process.^19^ In contrast, during the process of tumour metastasis and diffusion, cancer cells need to activate autophagy to obtain sufficient energy and promote EMT.^19^ The results of our Western blot analysis indicated that after Lar treatment, the LC3 II/I ratio was significantly elevated, demonstrating that Lar contributed to autophagosome formation and autophagic enhancement. Furthermore, we found that Lar decreased the expression of p62, an autophagic substrate, which is acknowledged as an autophagic flux indicator.^21^ Autophagic flux is a key intracellular process that plays an important role in maintaining human health.^22^ It has been demonstrated that decreased autophagic flux contributes to the development of cancer and may exacerbate chronic diseases such as dementia.^23^, ^24^, ^25^ Hence, exploring chemical reagents that activate autophagic flux may be useful for anti‐cancer therapy.^25^ Here, to the best of our knowledge, for the first time, Lar was found to activate autophagic flux, as evidenced by the presence of a higher number of red fluorescent puncta in Lar‐treated colon cancer cells than in control cells. Lar also increased the amount of LC3B‐II and decreased the expression of p62. In comparison, CQ, a late‐stage autophagy inhibitor that enhances autophagosome formation and suppresses autophagosome‐lysosome fusion,^22^ diminished the Lar‐induced accumulation of autolysosomes as well as cytotoxicity. Based on our findings, we propose that Lar‐induced autophagic cell death may be a valuable strategy to be used for CC cancer therapy.

Autophagy and apoptosis are under the control of multiple molecules and signalling pathways.^26^, ^27^ Classical regulation of autophagy is controlled by AMPK‐mediated inhibition of the mTOR pathway.^26^, ^27^ For instance, transient elevation of phosphorylated AMPK and reduction in phosphorylated mTOR induce autophagy in breast cancer cells.^27^ Consistent with these findings, we found that Lar increased AMPK phosphorylation and suppressed mTOR phosphorylation in colon cancer cells. Interestingly, when we transfected siAMPK to prevent AMPK activation in colon cancer cells in the presence of Lar, the Lar‐induced accumulation of autolysomes was significantly diminished in colon cancer cells. Furthermore, AMPK activation has also been shown to suppress EMT by elevating the expression of the epithelial marker E‐cad and decreasing the expression of mesenchymal markers in colorectal cancer.^28^ Activation of AMPK/mTOR signalling by irisin was also found to inhibit EMT by increasing E‐cad expression and decreasing vimentin expression in pancreatic cancer.^29^ We subsequently assessed the expression of EMT markers in colon cancer cells transfected with siAMPK in the presence of Lar. Interestingly, ablation of AMPK abolished Lar‐induced suppression of the EMT process. These data indicate that Lar activates autophagy and suppresses EMT in colon cancer cells through the AMPK/mTOR pathway.

In summary, the present study demonstrated that Lar‐induced cell death in colon cancer by inducing autophagic flux and suppressing EMT. Our study further indicates that the potential anti‐cancer effects of Lar in colon cancer may be achieved by activating AMPK/mTOR‐mediated autophagic cell death.

## 4 MATERIALS AND METHODS

### 4.1 Cell culture

Colon cancer cell lines, including COLO205 and HCT116, were purchased from the Cell Bank of the Chinese Academy of Sciences (Shanghai, China). COLO205 and HCT116 cells were cultured in Dulbecco's modified Eagle's medium (DMEM) (Gibco, USA) supplemented with 10% foetal bovine serum (FBS; Gibco, USA), and 100 units/ml penicillin and 100 μg/ml streptomycin (Gibco, USA). Human colonic mucosal epithelial cells (CP‐H040) were purchased from Procell (Wuhan City, China) and cultured in human colonic mucosal epithelial cells complete medium (CP‐H040, Procell, Wuhan City, China) supplemented with 10% foetal bovine serum (FBS; Gibco, USA), and 100 units/ml penicillin and 100 μg/ml streptomycin (Gibco, USA). The cells were cultured at 37°C in a humidified incubator containing 5% CO_2_ for further studies.

### 4.2 Cell viability assay

Cell viability was determined using a Cell Counting kit‐8 (CCK‐8, Beijing Solarbio Science & Technology Co., Ltd.). In brief, COLO205 and HCT116 cells (3 × 10^3^/well) were seeded in a 96‐well plate. Next, the cells were treated with 100, 200, 400, 800, 1600 and 3200 nM and 125, 250, 500, 1000, 2000 and 4000 nM Larotrectinib (Lar, ARRY‐470, Stelleck, https://www.selleck.cn/products/loxo‐101.html) for 24 h. Later, 10 μl CCK‐8 was added to each well for 1 h at 37°C. Optical density (OD) was determined using a Nanodrop microplate reader (Thermo) at 450 nm.

### 4.3 Transwell assay

COLO205 and HCT116 cells were cultured in 200 μl medium without FBS in the upper chamber (8 μm pore size chamber inserts; Corning, USA) for 24 h. The cells were then treated with or without 300 nM Lar for 24 h. Meanwhile, 500 μl of medium containing 10% FBS was added to the lower chamber. Subsequently, all the cells were maintained in an incubator at 37°C and 5% CO_2_ for another 48 h. The cells that migrated through the membrane were fixed with 4% paraformaldehyde (Beijing Solarbio Science & Technology Co., Ltd.) for 20 min, stained with 0.1% crystal violet (Beijing Solarbio Science & Technology Co., Ltd.) for 2 min, and washed with PBS to decrease the background. Images obtained from five random fields were observed under a microscope (Olympus Corporation, Tokyo, Japan).

### 4.4 Western blot

COLO205 and HCT116 cells or tumour tissues were lysed using a total protein extraction kit (Beijing Solarbio Science & Technology Co., Ltd.) with a protease inhibitor cocktail (Sigma, P8340). Protein concentration was determined using a BCA protein assay kit (Pierce; Thermo Fisher Scientific Inc.). All the samples (20 μg/lane) were subjected to 10% SDS‐PAGE. After electrophoresis, the proteins were transferred to polyvinylidene difluoride (PVDF) membranes (Pierce), and then blocked with 5% non‐fat dried milk in PBST (Beijing Solarbio Science & Technology Co., Ltd.) for 2 h at room temperature. Next, the membranes were incubated with primary antibodies overnight at 4°C. Primary antibodies against the following proteins and purchased from Cell Signalling Technology Inc were used: p‐AMPK (2537, 1:1000;), AMPK (5831, 1:1000), p‐mTOR (5536, 1:1000), mTOR (2983, 1:1000), LC3 (3868, 1:1000), p62 (39,749, 1:1000), E‐cadherin (3195, 1:1000), vimentin (5741, 1:1000), MMP2 (40,994, 1:1000), MMP9 (13,667; 1:1000) and GAPDH (5174, 1:4000). The membranes were then washed with PBST three times and incubated with horseradish peroxidase (HRP)‐conjugated goat anti‐rabbit IgG (1:5000; cat. no. ZB‐2301; Beijing Zhongshan Golden Bridge Biotechnology Co.) for 2 h at room temperature, followed by three washes with TBST. Immunoreactive protein bands were detected using enhanced chemiluminescence (EMD Millipore, Billerica, MA, USA). The relative protein expression was normalized to that of GAPDH. All experiments were repeated thrice. Image Lab™ software (Bio‐Rad, USA) was used for densitometric analysis.

### 4.5 RT‐PCR

SuperScript IV one‐step RT‐PCR system (12594100, ThermoFisher) was carried out to explore the effects of Lar on COLO205 and HCT116 cells according to the instructions. The primers used in the present study (5′–3′) were listed as follows:

E‐cad‐F: GGGGTTAAGCACAACAGCAA;

E‐cad‐R: CAAAATCCAAGCCCGTGGTG;

Vimentin‐F: GCTTCAGAGAGAGGAAGCCG;

Vimentin‐R: AAGGTCAAGACGTGCCAGAG;

MMP‐2‐F: GGCGGTCACAGCTACTTCTT;

MMP‐2‐R: GAGGGTTGGTGGGATTGGAG;

GAPDH‐F: GCCGTCTAGAAAAACCTGCC;

GAPDH‐R: ACCACCTGGTGCTCAGTGTA.

### 4.6 LC3 puncta

COLO205 and HCT116 cells were seeded in 6‐well plates in which cover glasses were placed, and the plates were incubated for 24 h at 37°C. The cells were then transfected with GFP‐mCherry‐LC3 adenovirus or GFP‐LC3 adenovirus vector (Hanheng Biotechonology Co. Ltd, https://www.hanbio.net/cn/services/9) for 24 h at 37°C. After treatment with 300 nM Lar or 10 μ0 BIX‐01294 (HY‐10587, MCE) at 37°C for 24 h, the cells were fixed with 4% paraformaldehyde (Beijing Solarbio Science & Technology Co. Ltd.) and analysed using confocal microscopy (Leica STELLARIS, Japan). Autophagy flux was determined by counting cells that displayed GFP+/mCherry+ (yellow, autophagosome) or GFP‐/mCherry+ (red, autolysosomes) puncta.

### 4.7 Wound healing assay

COLO205 and HCT116 cells (1 × 10^6^ cells/well) were seeded in 6‐well plates at 95% confluence. After 24 h, a linear scratch (representing a ‘wound’ in vitro) was created using a 200 μl sterile pipette tip across the monolayers. Cell debris was removed using PBS three times, and the adherent cells were incubated in DMEM with 2% FBS at 37°C under 5% CO_2_. After 24 h, the wounded monolayers were photographed under a microscope (Olympus Corporation, Tokyo, Japan). The remaining wound area was determined using Image Lab™ software (Bio‐Rad, USA).

### 4.8 Transmission electron microscope

COLO205 and HCT116 cells were collected, suspended and fixed overnight at 4°C in 2% glutaraldehyde (http://www.tianld.com/djhc) and 1% tannic acid (403,040, Sigma) prepared in 0.1 M sodium cacodylate (pH 7.3, C0250, Sigma). The cells were washed three times in sodium cacodylate buffer and incubated in 2% osmium tetroxide (http://www.tianld.com/djhc) for 2 h at room temperature. Next, the cells were washed thrice in ddH_2_O and exposed to 1% uranyl acetate (http://www.tianld.com/djhc) in water for 15 min at room temperature. Subsequently, the samples were dehydrated using an ascending ethanol series and embedded in Spurr's low‐viscosity medium (http://www.tianld.com/djhc). Ultrathin sections were cut using a Reichert Ultracut S ultramicrotome (Science Service, Munich, Germany) and contrasted with lead citrate. Images were viewed and captured using an EM 10 CR electron microscope (Carl Zeiss Gemini, Germany).

### 4.9 Transient transfection

siRNA oligonucleotides and negative control (NC) were purchased from GenePharma (China). Sequences of the siRNAs were as follows: NC (sense 5′–UUCUCCGAACGUGUCACG UTT–3′, antisense 5’–ACGUGACACGUUCGG AGAATT–3′), AMPK (sense 5′–AGUGAAGGUUGGCAAACAU–3′, antisense 5′–AUGUUUGCCAACCUUCACU–3′). Transfection was carried out using HiPerFect transfection reagent (Qiagen). In brief, COLO205 and HCT116 cells were seeded in a 6‐well plate at a density of 10^5^ cells/well for 24 h. HiPerFect transfection reagent (12 μl) was mixed with 100 μl serum‐free DMEM at room temperature, and 2 μl siRNA was added to the above mixture at a final concentration of 20 nM; this mixture was incubated for 10 min at room temperature. Subsequently, the mixture was added to the 6‐well plate for another 48 h, and the cells were collected for further analysis.

### 4.10 Immunofluorescence (IF)

COLO205 and HCT116 cells were seeded onto a cover slip placed in a 6‐well plate at a density of 10^5^ cells/well overnight. Next, the cells were treated with 300 nM Lar for 24 h. The cells were washed three times with PBS and incubated with primary antibodies against Ki67 (12,075, CST, 1:100) at 4°C overnight. After washing with PBST three times, the coverslips were blocked with 5% BSA at room temperature for 2 h. Next, the cells were incubated with goat anti‐rabbit IgG/FITC (SF134, Beijing Solarbio Science & Technology Co., Ltd.) at room temperature for 2 h. Fluorescence was observed under a fluorescence microscope (20× magnification; Olympus Corporation, Tokyo, Japan).

### 4.11 In vivo assay

Animal care and experimental procedures were approved by the Animal Ethics Committee of the Zhejiang University School of Medicine (ZJUMS‐2020Aj386).

Seven‐week‐old male nude mice (BALB/c) were purchased from Beijing Sipeifu Co. Ltd. (http://www.spf‐tsinghua.com/) and randomly divided into two groups (*n* = 5/group): (A) Control group (Con) and (B) Lar‐treated group. (A) Control HCT116 cells and (B) Lar‐treated HCT116 cells (1 × 10^6^ cells in 100 μl PBS each) were injected subcutaneously into the right posterior flank of the mice. When tumour sizes reached approximately >35 mm^3^, a preliminary experiment was carried out, and our data demonstrated that treatment with 20 mg/kg Lar (administered intragastrically, ig) twice daily for 3 weeks suppressed tumour growth in the mice. Hence, the mice in group B were treated with 20 mg/kg Lar twice daily for 3 weeks in the present study. Mice in group A were treated with an equal volume of vehicle. At the end of the 13‐week experiment, the mice were anaesthetised with pentobarbital sodium (50 mg/kg; intraperitoneal injection)^30^ and were sacrificed by cervical dislocation. Tumours were excised and weighed, and the tumour volume was calculated using the formula: tumour volume = 1/2 (length × width^2^).

### 4.12 Statistical analysis

All experiments were independently repeated at least thrice. Data are presented as mean ± SD. Unpaired Student's *t*‐test was performed to compare the means of two groups. One‐way analysis of variance (anova) followed by post hoc analysis was used for comparison among three or more groups. All the data were analysed using GraphPad Prism 8.0 (GraphPad Software, Inc.). A *p*‐value <0.05 was considered statistically significant.

## AUTHOR CONTRIBUTIONS

**Shenglin Ma:** Conceptualization (equal); data curation (equal); formal analysis (equal); funding acquisition (equal); investigation (equal); methodology (equal); project administration (equal); resources (equal); software (equal); supervision (equal); validation (equal); visualization (equal); writing – original draft (equal); writing – review and editing (equal). **Wencheng Kong:** Conceptualization (equal); data curation (lead); formal analysis (lead); investigation (lead); methodology (lead); resources (lead); validation (lead); writing – original draft (equal); writing – review and editing (equal). **Hangzhang Zhu:** Data curation (equal); investigation (equal); software (equal). **Sixing Zheng:** Data curation (equal); software (equal); validation (equal). **Guang Yin:** Data curation (equal); investigation (equal). **Panpan Yu:** Methodology (equal); supervision (equal). **Yuqiang Shan:** Methodology (equal); software (equal). **Xinchun Liu:** Methodology (equal); software (equal). **Rongchao Ying:** Formal analysis (equal). **Hong Zhu:** Resources (equal).

## CONFLICT OF INTEREST

The authors report no conflicts of interest.

## Data Availability

The data that support the findings of this study are available from the corresponding author upon reasonable request.
